# Different perception of dry eye symptoms between patients with and without primary Sjogren’s syndrome

**DOI:** 10.1038/s41598-022-06191-x

**Published:** 2022-02-09

**Authors:** Minjeong Kim, Yeoun Sook Chun, Kyoung Woo Kim

**Affiliations:** 1 Department of Ophthalmology, Chung-Ang University College of Medicine, 102 Heukseok-ro, Dongjak-gu, Seoul, 06973 Republic of Korea; 2 Biomedical Research Institute, Chung-Ang University Hospital, Seoul, Republic of Korea

**Keywords:** Eye manifestations, Eye diseases

## Abstract

Here, we investigated the different perception of dry eye symptoms between in patients with and without primary Sjogren’s syndrome (pSS). In this study, 221 patients with dry eye disease (DED) without pSS (non-SS DED group) and 55 patients with DED with pSS (SS DED group) were included. The ocular discomfort was evaluated using ocular surface disease index (OSDI) questionnaire and patients were further divided into 3 severity subgroups according to OSDI scores. The OSDI score was higher in the non-SS DED group even after matching corneal erosion scores despite the ocular surface erosions and tear deficiency was worse in the SS DED group. The corneal sensitivity was nearly normal in both groups without inter-group difference (Non-SS DED group: 5.82 ± 0.54 cm, SS DED group: 5.90 ± 0.29 cm, *p* = 0.217). Moreover, all clinical parameters were not significantly correlated with OSDI scores in both non-SS DED group and SS DED group. In the mild and severe OSDI subgroups, the ocular surface erosions and tear deficiency were worse in the SS DED group whereas the OSDI scores were not different between groups. In conclusion, clinicians should be aware that pSS patients may complain less of their discomfort unlike their actual severe status of DED.

## Introduction

Dry eye disease (DED) is a common, worldwide ocular surface disease that deteriorates quality of daily lives^1,2^. Although there are several parameters suggested to evaluate this multifactorial inflammatory disease, there has been no definite consistency between signs and symptoms^3–5^. Therefore, detailed assessment of subjective symptoms is needed to fulfill patients’ needs in dealing with DED^6^. Various questionnaires to properly measure patients’ experience of dry eye symptoms were devised. One of them is ocular surface disease index (OSDI), which is a 12-item scale composed of 3 subscales including patient’s symptoms, functional discomfort in daily lives and exacerbating environmental conditions^7^. The total score is calculated and divides patients into normal, mild, moderate and severe groups. As a well-known indicator that converts subjective discomfort into objective numbers, it showed meaningful correlation with a dry eye parameter^8^ with significant test reliability and validity^9^ and has been applied to assess clinical symptoms of DED^10,11^ and result of interventions^12,13^ in various clinical studies.

In clinical settings, DED patients with similar levels of objective dry eye parameters may complains different levels of discomfort and vise versa^3^. The multifactorial pathogenesis of DED can partly explain this discrepancy but considering that the cornea is a densely innervated organ, we hypothesized each patient may have different sensory nervous function or neurogenic inflammation in perceiving ocular discomfort.

Primary Sjogren’s syndrome (pSS) is a chronic autoimmune disease that has a unique pathophysiology. It affects the exocrine function of the lacrimal and salivary glands, and patients also suffer from peripheral neuropathy, including that of the corneal nerve. Structural differences in corneal nerves and the unique distribution of immune cells were found in some patients, and it was suggested that these changes might explain the altered corneal sensitivity^14^. Accordingly, the hyperexcitability of peripheral neurons and further high order neurons to evoke central sensitization without corneal stains may be induced by neuropathic pain in DED^15,16^. On the other hands, however, the TRP cation channel subfamily V member 1 (TRPV1) which is activated by hyperosmolarity^17^ and is important for sensory transduction in polymodal nociceptors^18^ became rather desensitized or inhibited by the prolonged or repeated activation^19^.

Herein, we compared the subjective perception of dry eye symptoms between DED with and without pSS to understand how the chronic ocular surface inflammation as seen in pSS contributes to the level of ocular discomfort.

## Results

### Demographics and clinical parameters of DED in patients with and without pSS

A total of 276 eyes from 276 patients with DED in accordance with the Dry Eye Workshop (DEWS) II criteria were included. The 221 patients were with DED without pSS (i.e., non-SS DED) group and the other 55 patients were with DED with pSS (i.e., SS DED) group. The average age in each group was 58.5 ± 15.4 and 54.4 ± 12.9 years, respectively, which was not significantly different between the two groups (Table 1). The percentage of females was higher in the SS DED group (94.5% vs. 73.3% in the non-SS DED group, *p* = 0.001, Table 1), which was similarly reported in a previous report that showed that the ratio of females to males in pSS was 16:1^20^.

**Table 1:** Demographics of dry eye disease (DED) patients with and without primary Sjogren’s syndrome (SS).

Variables	Group		*p* value
	Non-SS DED	SS DED	
No. of patients/eyes	221/221	55/55	–
**Demographics**			
Age	58.5 ± 15.4	54.4 ± 12.9	0.072
Male/female (% female)	59/162 (73.3%)	3/52 (94.5%)	0.001*
OSDI (score)	43.6 ± 21.1	36.3 ± 20.5	0.022*
**Clinical parameters of DED**			
Tear MMP-9 (Gr)	1.68 ± 1.10	1.78 ± 1.09	0.452
Tear osmolarity (mOsm/L)	315.5 ± 28.4	313.7 ± 21.4	0.699
SICCA OSS	1.42 ± 2.08	3.06 ± 2.44	< 0.001*
Tear secretion (mm)	11.3 ± 8.1	7.4 ± 6.8	< 0.001*
Corneal sensitivity (cm)	5.82 ± 0.54	5.90 ± 0.29	0.217
Corneal erosion score	1.76 ± 2.27	2.45 ± 2.02	0.006*
Tear BUT (s)	5.76 ± 2.06	5.39 ± 2.25	0.173
MG expressibility (Gr)	1.49 ± 0.66	1.46 ± 0.57	0.694
Meibum quality (Gr)	1.57 ± 0.77	1.46 ± 0.81	0.190

*OSDI* ocular surface disease index, *MMP-9* matrix metalloproteinase 9, *Gr* grade, *OSS* ocular staining score, *BUT* break-up time, *MG* meibomian gland.

*p < 0.05.

Significant values are in bold.

**Figure 1:**
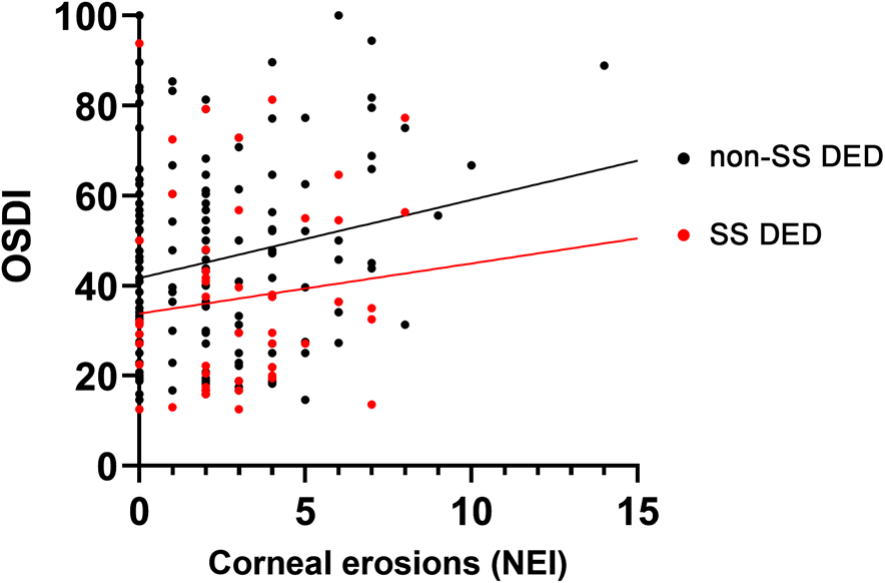
Scatter plots showing the OSDI scores according to the corneal erosion scores in dry eye disease patients with primary Sjogren’s syndrome (SS DED group) and without primary Sjogren’s syndrome (non-SS DED group). A regression line for the mean scatter plot of analysis of covariance is higher in the non-SS DED group than in the SS DED group.

The OSDI score was significantly higher in the non-SS DED group (43.6 ± 21.1) than in the SS DED group (36.3 ± 20.5, *p* = 0.022, Table 1). Among the clinical parameters of DED, Sjogren’s International Collaborative Clinical Alliance (SICCA) ocular staining score (OSS), tear secretion by Schirmer I without anesthesia and corneal erosion scores were higher in the SS DED group (*p* < 0.001, *p* < 0.001 and *p* = 0.006, respectively, Table 1). There was no significant difference in the threshold of corneal sensitivity between the two groups (Table 1). The OSDI score in non-SS DED group was still higher than in SS DED group after matching the corneal erosion scores (*p* = 0.004, Fig. 1).

### Clinical parameters of DED according to the severity of OSDI score in patients with and without pSS

**Figure 2:**
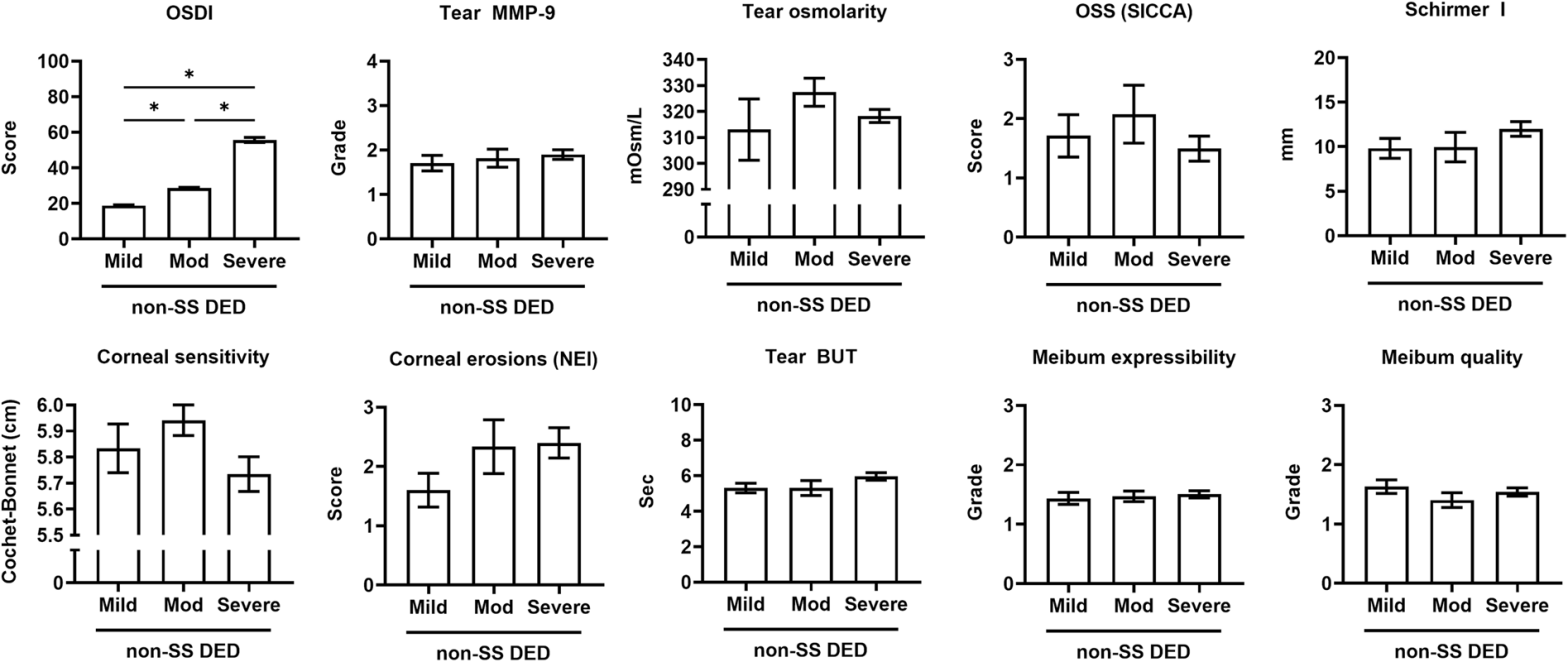
Differences in clinical parameters of dry eye disease (DED) among the OSDI severity subgroups in the patients without primary Sjogren’s syndrome (non-SS DED group). None of the DED parameters showed significant differences among subgroups. *p < 0.05. Mean ± standard error measurement.

**Figure 3:**
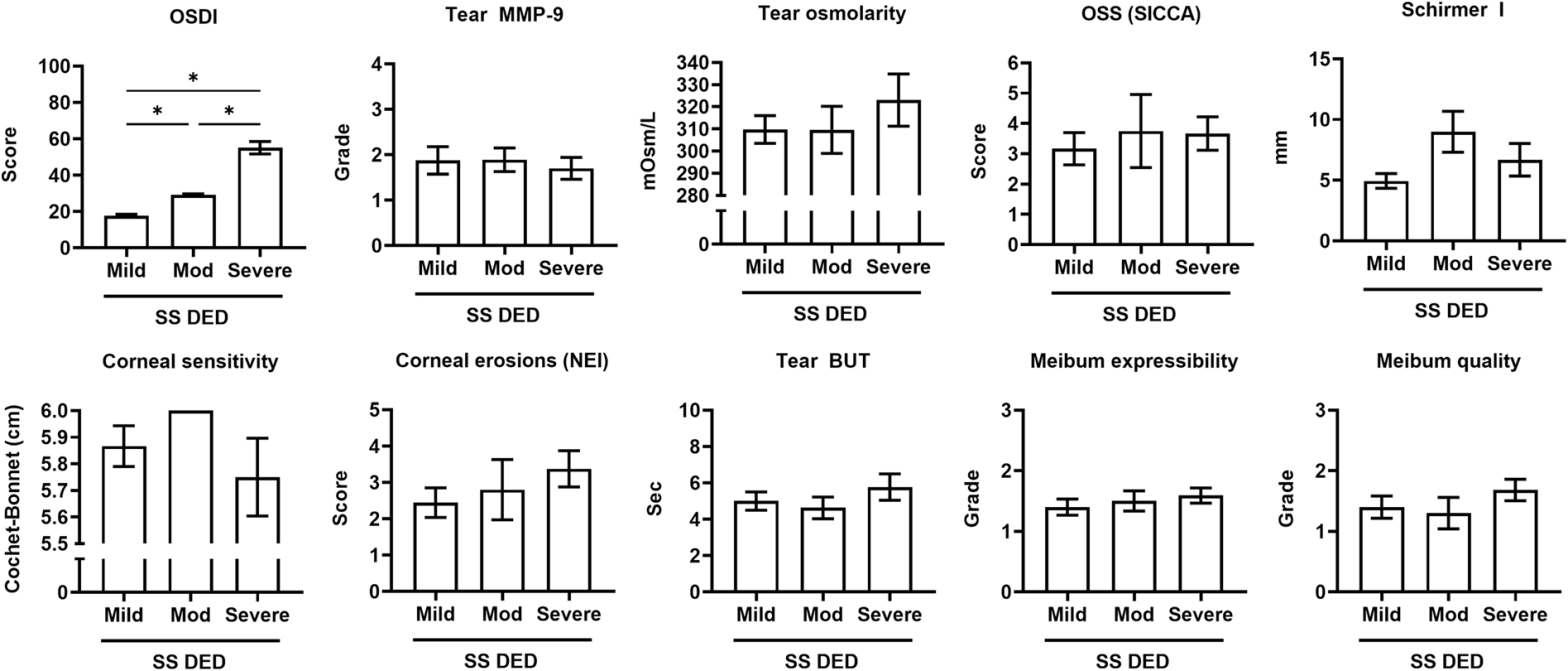
Differences in clinical parameters of dry eye disease (DED) among the OSDI severity subgroups in the patients with primary Sjogren’s syndrome (SS DED group). The difference in tear secretion by the Schirmer I test between the mild and moderate OSDI severity subgroups was significant. *p < 0.05. Mean ± standard error measurement.

All clinical parameters of DED were not different among OSDI severity subgroups in the non-SS DED group (Fig. 2) and in the SS DED group (Fig. 3).

### Correlation of OSDI scores with the clinical parameters of DED in patients with and without pSS

The relationship between the OSDI score and the clinical parameters for DED was analyzed with Spearman’s rank correlation test to identify contributing factors to subjective dry eye symptoms. All independent DED parameters as well as age were not correlated with the OSDI scores in both non-SS DED group (Table 2) and SS DED group (Table 3).

**Table 2:** Correlations of the ocular surface disease index (OSDI) scores with values of age and parameters for dry eye disease (DED) in patients with non-Sjogren’s syndrome (non-SS) DED.

Non-SS DED group	OSDI score vs	
Variables	Correlation coefficient	*P*
Age (years)	0.042	0.536
Tear MMP-9 (Gr)	0.080	0.283
Tear osmolarity (mOsm/L)	− 0.096	0.295
SICCA OSS	− 0.080	0.325
Tear secretion (mm)	0.130	0.085
Corneal sensitivity (cm)	− 0.131	0.129
Corneal erosion score	0.102	0.188
Tear BUT (s)	0.128	0.104
MG expressibility (Gr)	0.017	0.804
Meibum quality (Gr)	− 0.011	0.881

*MMP-9* matrix metalloproteinase 9, *Gr* grade, *OSS* ocular staining score, *BUT* break-up time, *MG* meibomian gland.

*p < 0.05.

**Table 3:** Correlations of the ocular surface disease index (OSDI) scores with values of age and parameters for dry eye disease (DED) in patients with Sjogren’s syndrome (SS) DED.

SS DED group	OSDI score vs	
Variables	Correlation coefficient	*P*
Age (years)	− 0.229	0.199
Tear MMP-9 (Gr)	− 0.094	0.539
Tear osmolarity (mOsm/L)	0.261	0.266
SICCA OSS	0.029	0.846
Tear secretion (mm)	0.123	0.448
Corneal sensitivity (cm)	− 0.021	0.900
Corneal erosion score	0.128	0.365
Tear BUT (s)	0.127	0.461
MG expressibility (Gr)	0.222	0.114
Meibum quality (Gr)	0.182	0.198

*MMP-9* matrix metalloproteinase 9, *Gr* grade, *OSS* ocular staining score, *BUT* break-up time, *MG* meibomian gland.

*p < 0.05.

### Differences in clinical parameters of DED between the patients with and without pSS in each OSDI severity subgroup

In the mild and severe OSDI subgroups, although the SICCA OSS or the corneal erosion score was higher and tear secretion was lower in the SS DED group, there was no significant difference in the OSDI score between the two groups (Table 4). In the moderate OSDI subgroup, no clinical parameters of DED or OSDI scores were distinguishable between the two groups (Table 4).

**Table 4:** Difference of the clinical parameters of dry eye disease (DED) between in patients with primary Sjogren’s syndrome (SS) DED and non-SS DED in each severity group of the ocular surface disease index (OSDI) score.

Variables	OSDI severity subgroups								
	Mild			Moderate			Severe		
	Non-SS DED	SS DED	*p* value	Non-SS DED	SS DED	*p* value	Non-SS DED	SS DED	*p* value
No. of patients/eyes	49/49	20/20	-	32/32	11/11	-	140/140	24/24	-
OSDI score	18.7 ± 2.6	17.7 ± 3.4	0.184	28.6 ± 2.5	29.1 ± 2.1	0.613	55.7 ± 16.8	55.1 ± 16.9	0.861
Tear MMP-9 (Gr)	1.7 ± 1.1	1.9 ± 1.2	0.622	1.8 ± 1.0	1.9 ± 0.8	0.846	1.9 ± 1.2	1.7 ± 1.1	0.491
Tear osmolarity (mOsm/L)	313.0 ± 60.3	309.8 ± 17.7	0.122	327.4 ± 21.6	309.6 ± 23.7	0.131	318.3 ± 22.2	323.0 ± 31.2	0.606
SICCA OSS	1.7 ± 2.0	3.2 ± 2.3	0.016*	2.1 ± 2.5	3.8 ± 3.4	0.226	1.5 ± 2.1	3.7 ± 2.5	< 0.001*
Tear secretion (mm)	9.8 ± 6.9	4.9 ± 2.5	0.001*	10.0 ± 7.9	9.0 ± 4.5	0.781	12.0 ± 8.8	6.7 ± 5.4	0.002*
Corneal sensitivity (cm)	5.8 ± 0.5	5.9 ± 0.3	0.964	5.9 ± 0.2	6.0 ± 0.0	1.000	5.7 ± 0.6	5.8 ± 0.5	0.984
Corneal erosion score	1.6 ± 1.6	2.4 ± 1.7	0.088	2.3 ± 2.2	2.8 ± 2.6	0.600	2.4 ± 2.7	3.4 ± 2.4	0.033*
Tear BUT (s)	5.3 ± 1.7	5.0 ± 2.0	0.352	5.3 ± 2.0	4.6 ± 1.7	0.404	6.0 ± 2.2	5.8 ± 2.6	0.770
MG expressibility (Gr)	1.4 ± 0.7	1.4 ± 0.6	0.845	1.5 ± 0.5	1.5 ± 0.5	1.000	1.5 ± 0.7	1.6 ± 0.6	0.580
Meibum quality (Gr)	1.6 ± 0.8	1.4 ± 0.8	0.278	1.4 ± 0.7	1.3 ± 0.8	0.696	1.5 ± 0.8	1.7 ± 0.8	0.440

*MMP-9* matrix metalloproteinase 9, *Gr* grade, *OSS* ocular staining score, *BUT* break-up time, *MG* meibomian gland.

*p < 0.05.

Significant values are in bold.

## Discussion

DED is a complicated, multifactorial disease in which various ocular conditions work together to form a vicious cycle. Reduced tear production from the lacrimal gland or excessive evaporation on the ocular surface results in high tear osmolarity, which in turn initiates the inflammatory cascade. Tear osmolarity itself and the induced inflammation collaborate to cause damage to goblet cells and epithelial cells, which exacerbates ocular inflammation and increases tear instability that amplifies tear hyperosmolarity and restarts the vicious cycle of DED^21^. This complicated feature of DED explains the result that there were few parameters related to the severity of symptoms. In the present study, we investigated the different perceptions of dry eye symptoms between patients with and without pSS based on the OSDI scores. We revealed that OSDI scores were even higher in patients without pSS than in age- and period-matched patients with pSS, although the clinical parameters of DED were even worse in patients with pSS.

In both non-SS DED group and SS DED group, the OSDI score was not significantly correlated with the corneal staining scores. Despite the sensation of discomfort may be multifactorial, we hypothesized that the corneal nerve became adapted to chronic ocular stimulations in DED patients regardless of the comorbidity of pSS. In fact, this hypothesis has been proposed previously. For example, wearing a contact lens for a long time induced sensory adaptation and lower corneal sensitivity^22^, which recovered after not using those lenses^23^. In addition, Xu et al. demonstrated that dry eye patients have lower sensitivity because they have higher staining scores^24^. This adaptation of the corneal nerve leads to hyposensitivity, which may decrease ordinary tear secretion and further cause more erosions, which in turn irritate the cornea, making vicious.

Unexpectedly, the thresholds of corneal sensitivity according to Cochet-Bonnet esthesiometer were not significantly different between two groups and the average values were even nearly normal. However, we cannot be sure if the patients in both groups had normal density of peripheral corneal nerve because in vivo confocal analysis was absent in this study. In fact, it was reported that the modified Belmonte non-contact esthesiometer^14^ and Cochet-Bonnet esthesiometer^25^ did not reflect the morphological damage of corneal nerves in patients with pSS. A future study needs to verify the difference of OSDI scores according to the possible difference of the morphological features of corneal nerve with versus without pSS.

In subgroup analysis according to OSDI severe scores, the moderate severity group did not reveal the difference of ocular surface erosions and tear production between the Non-SS DED group and SS DED group unlike in mild and severe subgroups. We thought that this was because the moderate severity group included patients with higher tear production relatively more compared to other subgroups. This might be due to the selection bias that occurred with a small number of patients or due to the reflex tearing which could not occur in patients with severely damaged lacrimal gland.

When eye dryness occurs, extracellular matrix (ECM) likes to decrease in the intercellular space in the corneal epithelium, thereafter the thinning of the ECM contributes to enhanced friction and rigidity^26^. The irritation of corneal nerves by constantly enhanced shear in the intercellular space may result in the neurogenic inflammation^26^. Maintained mechanical provocation produces sustained discharge of the polymodal nociceptors having a threshold slightly lower than pure mechano-nociceptors^18,26^. Specifically, DED eyes might have released neuropeptides such as substance P and calcitonin gene related peptide (CGRP) that induces neurologic inflammation, which make nerve to be sensitive to local inflammatory mediators leading to intermittent hypersensitivity to ocular pain or irritation^27^.

By contrast, it becomes more complicated in regard to pSS. Some recent studies demonstrated morphological changes in the corneal nerves of pSS patients^14,28,29^ which might be related to altered corneal sensitivity. There have been evidences to support the possible immunogenic injury that occurs in patients with pSS-related DED. This change was accompanied by more dendritic antigen-presenting cells in the center of the cornea^14^, which play an important role in the immune cascade by presenting target antigens to other immune cells. Additionally, there were more mature forms of dendritic cells in the central cornea of pSS patients, which means that active immune reactions took place. Although it is not fully understood how the autoimmune reaction of SS affects peripheral nerves, cytokines secreted by T lymphocytes and dendritic cells are crucial, as these cytokines induce inflammatory infiltration, causing vasculitis and nerve damage^30^. Nevertheless, the OSDI scores were even lower in the SS DED group than in the age- and period-matched non-SS DED group, which corresponds to results of a previous study that showed the lesser symptoms than signs in the presence of pSS or graft-versus-host disease^7^. Furthermore, the clinical parameters of DED were clearly worse in the SS DED group under the equal level of OSDI scores. As a possible mechanism, more severe ocular surface erosions and the resultant more chronic or prolonged neurogenic inflammation in dry eyes with pSS might have induced compensatory stress-relieving signals both physically and psychologically. In general, the stimulation of TRPV1 leads to a descending neuronal impulse or axon reflex, causing neuropeptide release from nearby nerve terminals to produce nociception^31^. In contrast, polymodal nociceptors may become rather desensitized or inhibited by prolonged or repeated activation^19^. We speculate that the diminished ocular surface discomfort in patients with pSS may be a result of the downregulation of dorsal root ganglion and peripheral expression of substance P and CGRP in the cornea. However, such a downregulation hypothesis may depend on the disease duration of DED. Future studies with disease duration-matched subjects will be needed to verify the hypothesis.

In a previous study, the DED symptoms according to OSDI scoring system and depressive symptom scores by Beck Depression Inventory were linearly correlated well^32^. Given that high scores of anxiety and depression was established in pSS patients^33^, the lower OSDI score in pSS patients seen in our study is an unexpected result. We suggest that the disease activity and severity of pSS is important to reveal discomfort in DED patients. Although we could not show the chronicity of pSS quantitatively in our cohort, the similar level of tear matrix metalloproteinase (MMP)-9 in the SS DED group compared with the non-SS DED group (Table 1) suggests that the cohort of SS DED in this study is in the steady state of ocular surface inflammation.

Our results correspond to a previous study by Mizuo et al.^34^, which showed worse signs of Schirmer testing, fluorescein staining, and rose bengal staining corneal erosions in patients with pSS than without pSS, whereas subjective symptoms were similar based on by 25-item National Eye Institute Visual Functioning Questionnaire (NEI-VFQ-25) and Medical Outcomes Study 8-item Short-Form Health Survey^34^. We evaluated the symptoms using OSDI scores and analyzed signs and symptoms in each OSDI severity subgroup. Furthermore, we attempted to correlate various signs, including tear MMP-9, tear osmolarity, corneal sensitivity, meibomian gland dysfunction grades, and tear break up time (BUT), as well as Schirmer testing and ocular surface staining scores.

The limitation of this study is that it is cross-sectional rather than longitudinal. Moreover, we could not prove the change in corneal nerve density or the anatomical morphology of the corneal nerve in pSS. In fact, after invasive ocular surgery that damages the corneal nerve, symptoms and objective signs changed with the passage of time according to previous studies^27^. Further research regarding the longitudinal alteration of the subjective ocular discomfort and corneal staining score as well as the corneal sensitivity in pSS patients would contribute to assessing the origin of ocular discomfort in pSS. Although we included various parameters of DED for analysis in subjects, the order of all those examinations should be ideal to obtain accurate test results. In this study, Schirmer I test should have been performed after the examination of BUT and ocular surface staining score to prevent the unexpected overestimation of conjunctival erosions possibly scratched by Schirmer strip papers. Nevertheless, this study has significant importance in that it is the first to focus and compare symptomatic differences in DED between non-SS and pSS patients. An advanced understanding of symptomatology in DED beyond clinicians’ observation would further improve patients’ quality of life.

Unfortunately, we evaluated DED-related subjective discomfort only with the OSDI scoring system in this study. However, several studies have reported that the symptoms in patents with DED were poorly associated with the objective clinical parameters probably due to the variability in pain and the variations of the disease process of DED^35–39^. To validate the different perception of dry eye symptoms according to the comorbidity of pSS more precisely, is may be required to use various questionnaires including NEI-VFQ-25, Standard Patient Evaluation of Eye Dryness Questionnaire, Dry Eye Questionnaire, Impact of Dry Eye in Everyday Life, Symptom Assessment In Dry Eye though additional studies.

In conclusion, the perception of dry eye symptoms was different between age- and period-matched patients with and without pSS. Relatively low OSDI scores and the feature of ocular discomfort in pSS, which was irrelevant to corneal sensitivity, suggest that chronic and prolonged inflammatory stimulation in the ocular surface may paradoxically induce discomfort-relieving signals in pSS. Therefore, it would be important to pay attention to pSS patients, as their clinical parameters of DED might be worse when they perceive fewer symptoms.

## Methods

This study was a retrospective cross-sectional comparative cohort study. The whole process properly followed the tenets of the Declaration of Helsinki. The study was approved by the Chung-Ang University Hospital Institutional Review Board (IRB) and the informed consent was waived by an IRB (Approval No. 2002-004-19301).

### Patients

Patients who satisfied the diagnostic criteria of DED established by the Tear Film and Ocular Surface Society DEWS II from October 2019 until November 2020 were included. They were selected by screening with symptomology using an OSDI score ≥ 13 and confirmed to have DED when fulfilling one of the following criteria: noninvasive tear breakup time < 10 s, tear osmolarity ≥ 308 mOsm/L in either eye or interocular difference > 8 mOsm/L, ocular surface staining > 5 corneal spots, > 9 conjunctival spots, or lid margin (≥ 2 mm length & ≥ 25% width)^40^.

Enrollees were divided into two groups: SS DED and non-SS DED. All patients with SS DED were diagnosed with SS in accordance with the 2016 American College of Rheumatology (ACR)/European League Against Rheumatism (EULAR) classification criteria^41^. Three points were assigned to biopsy and immunology: labial salivary gland with focal lymphocytic sialadenitis & focus score of ≥ foci/4 mm^2^ and anti-SSA/Ro-positive. The other items weighed 1 point: ocular staining score ≥ 5 in at least one eye, Schirmer’s test ≤ 5 mm/5 min in at least one eye, unstimulated whole saliva flow rate ≤ 0.1 mL/min. If the sum of all categories was 4, larger patients were diagnosed with pSS.

### OSDI questionnaire

The enrollees were asked to fill out OSDI questionnaire to assess the subjective ocular symptoms of dry eye disease and its effect in vision-related function. The survey was targeted for a period of one week before the day of the survey and was composed of 3 subscales: ocular symptoms, vision-related daily function and environmental triggers. Patients answered twelve questions in total with scale from 0 to 4, 0 corresponding to “none” and 4 corresponding to “always”. Sum of scores multiplied by 25 were divided with the number of questions properly answered to calculated OSDI score. The patients in both each group were further classified into three subgroups according to the OSDI severity: mild (i.e. 13 to 22), moderate (i.e. 23 to 32) and severe (i.e. 33 and more)^42^.

### Clinical parameters to evaluate DED

For objective assessment of DED, corneal sensitivity score using esthesiometer, tear osmolarity, tear MMP-9, tear secretion with Schirmer I without anesthesia, tear BUT, SICCA OSS, corneal erosion score according to the National Eye Institute/Industry (NEI) grading scale, meibomian gland (MG) expressibility and meibum quality of the secreted meibum were evaluated. The examination was performed in order of listed.

Assessment for corneal sense was performed with a Cochet-Bonnet esthesiometer (Luneau ophthalmology, Chartres Cedex, France). Starting from the longest length, 6 cm, the test was performed by decreasing the length of the monofilament that touches the center of the cornea by 0.5 cm until the patient first felt discomfort.

To measure tear osmolarity, a test strip of I-PEN™ (I-MED Pharma Inc., Montreal, QC, Canada) was soaked with tear at the lower conjunctival fornix and assembled into an analyzer that shows test results in digits. The test for tear MMP-9 was performed with a point-of-care immunoassay kit (InflammaDry®, Quidel, San Diego, CA, USA), which was previously demonstrated to properly diagnose inflammation related to dry eye, following instructions from product documentation^43^. A sterile sample collector dabbed multiple areas along the lower palpebral conjunctiva to collect the tear fluid and assembled into the immunoassay test cassette. After 20 s of activation in buffer solution, the intensity of the red line in a readout window was scored from grade 0 to grade 4 based on a 5-stage grading system composed of negative, trace, weak positive, positive and strong positive^44^.

The Schirmer I test was used to evaluate tear secretion. A Schirmer standard strip (Eagle Vision, Memphis, TN, USA) was applied on the outer 1/3 point of the lower conjunctival fornix, and tear fluid was absorbed for 5 min. No analgesic eyedrops were instilled.

After more than 15 min from Schirmer I test, the BUT was measured as previously established^45^. After a drop of normal saline was put on strip paper coated with fluorescein dye (Haag-Streit international, Koniz, Switzerland) and the saline was then shaken off. The strip was gently applied to the lower lid margin and stained tear film. The time when the first break of tear film was observed under a cobalt blue filter after the last blinking was considered the BUT. Thereafter, the ocular staining score was evaluated. Each eye underwent slit lamp examination under a yellow filter after fluorescein instillation^46^. Both the SICCA score^47^ and NEI score^6^ were obtained following the established standard.

Evaluation for MG dysfunction (MGD) was performed in two ways: MG expressibility of 5 glands of the central upper lid and the quality of secreted meibum. MG expressibility of meibum from five glands with firm digital pressure was graded from 0 to 3: 0 = all glands expressible, 1 = 3–4 glands expressible, 2 = 1–2 glands expressible, 3 = no glands expressible and quality of meibum from 0 to 3, and quality of meibum from 0 to 3, each corresponding to clear, cloudy, cloudy particulate fluid and tooth-paste like according to previously demonstrated benchmarks^48^.

### Statistical analysis

For statistical analysis, SPSS software version 20.0 (SPSS, Inc., Chicago, IL, USA) and Prism software v.8.4.3 (GraphPad, La Jolla, CA, USA) were used. To compare each parameter between non-SS DED and SS DED groups, Student’s *t*-test was used. The comparison of OSDI scores between two groups after matching corneal erosion scores was analyzed by analysis of covariance (ANCOVA). Spearman’s rank correlation test was performed to analyze the correlation of OSDI scores with age and DED parameters. To assess the difference in each DED parameter in the 3 subgroups, one-way analysis of variance (ANOVA) with *Bonferroni*’s post hoc analysis was applied. The average and standard deviation of the data sets are expressed as ± and all statistical analyses were considered significant at *P* < 0.05.
